# Human dental pulp stem cell responses to different dental pulp capping materials

**DOI:** 10.1186/s12903-021-01544-w

**Published:** 2021-04-26

**Authors:** Chawan Manaspon, Chavin Jongwannasiri, Sujin Chumprasert, Noppadol Sa-Ard-Iam, Rangsini Mahanonda, Prasit Pavasant, Thantrira Porntaveetus, Thanaphum Osathanon

**Affiliations:** 1 Dental Stem Cell Biology Research Unit, Faculty of Dentistry, Chulalongkorn University, Bangkok, 10330 Thailand; 2 Biomedical Engineering Institute, Chiang Mai University, Chiang Mai, 50200 Thailand; 3 Faculty of Medicine and Public Health, HRH Princess Chulabhorn College of Medical Science, Chulabhorn Royal Academy, Bangkok, 10210 Thailand; 4 Oral Biology Research Center, Faculty of Dentistry, Chulalongkorn University, Bangkok, 10330 Thailand; 5 Immunology Research Center, Faculty of Dentistry, Chulalongkorn University, Bangkok, 10330 Thailand; 6 Department of Periodontology, Faculty of Dentistry, Chulalongkorn University, Bangkok, 10330 Thailand; 7 Department of Anatomy, Faculty of Dentistry, Chulalongkorn University, Bangkok, 10330 Thailand; 8 Genomics and Precision Dentistry Research Unit, Department of Physiology, Faculty of Dentistry, Chulalongkorn University, Bangkok, 10330 Thailand

**Keywords:** Human dental pulp cells, Pulp capping, Mineral trioxide aggregate, Calcium silicate materials, Calcium hydroxide

## Abstract

**Background:**

Direct pulp capping is a vital pulp therapy for a pin-point dental pulp exposure. Applying a pulp capping material leads to the formation of a dentin bridge and protects pulp vitality. The aim of this study was to compare the effects of four dental materials, DyCal^®^, ProRoot^®^ MTA, Biodentine™, and TheraCal™ LC in vitro.

**Methods:**

Human dental pulp stem cells (hDPs) were isolated and characterized. Extraction medium was prepared from the different pulp capping materials. The hDP cytotoxicity, proliferation, and migration were examined. The odonto/osteogenic differentiation was determined by alkaline phosphatase, Von Kossa, and alizarin red s staining. Osteogenic marker gene expression was evaluated using real-time polymerase chain reaction.

**Results:**

ProRoot^®^ MTA and Biodentine™ generated less cytotoxicity than DyCal^®^ and TheraCal™ LC, which were highly toxic. The hDPs proliferated when cultured with the ProRoot^®^ MTA and Biodentine™ extraction media. The ProRoot^®^ MTA and Biodentine™ extraction medium induced greater cell attachment and spreading. Moreover, the hDPs cultured in the ProRoot^®^ MTA or Biodentine™ extraction medium migrated in a similar manner to those in serum-free medium, while a marked reduction in cell migration was observed in the cells cultured in DyCal^®^ and TheraCal™ LC extraction media. Improved mineralization was detected in hDPs maintained in ProRoot^®^ MTA or Biodentine™ extraction medium compared with those in serum-free medium.

**Conclusion:**

This study demonstrates the favorable in vitro biocompatibility and bioactive properties of ProRoot^®^ MTA and Biodentine™ on hDPs, suggesting their superior regenerative potential compared with DyCal^®^ and TheraCal™.

**Supplementary Information:**

The online version contains supplementary material available at 10.1186/s12903-021-01544-w.

## Background

The ultimate aim of restorative dentistry is to preserve dental pulp tissue vitality. Various therapeutic applications have been introduced to maintain dental pulp function. Direct pulp capping is performed to treat a pin-point dental pulp tissue exposure when there is no inflammation. In this method, a bioactive material is placed directly over the exposed dental pulp tissue. Pulp capping materials function as a physical barrier to seal and prevent a connection between the dental pulp and oral cavity, reducing potential irritation and infection. The material simultaneously promotes dental pulp healing by inducing reparative dentin. Moreover, the direct pulp capping method is more cost-effective compared with traditional root canal treatment [1].

For pulp wound healing process, dental pulp cells generally proliferate and also migrate to the injured site. Subsequently, these cells differentiate into odontoblast-like cells that form tertiary dentin [2]. The cells participating in this regeneration and repair processes originate from several locations in the pulp tissue, including the perivascular area [3]. In response to a deep dental cavity, the cells in the perivascular area proliferate, then migrate to an area adjacent to the injury site after 4–6 weeks, and form reactionary dentin [3]. Human dental pulp stem cells (hDPs) have been reported as a critical key for reparative dentin formation. STRO1-positive hDPs express pericyte-associated antigen, confirming the perivascular niche of hDPs [4]. These cells exhibit a fibroblast-like morphology with superior proliferative ability compared with human bone marrow-derived mesenchymal stem cells [5]. hDPs express mesenchymal stem cell markers and have multipotential differentiation ability [5]. It has been shown that hDPs can differentiate into ectodermal, mesodermal, and endodermal-derived cells [6–8]. Hence, these cells are proposed as an alternative cell source for various regenerative applications [9].

An ideal pulp capping material should prevent bacterial infiltration, trigger minimal inflammation, and induce dentin bridge formation. Current materials clinically used for pulp capping can be generally divided into calcium hydroxide (Ca(OH)_2_)-, mineral trioxide aggregate (MTA)-, calcium silicate-, and adhesive-based materials. A systematic review and meta-analysis demonstrated that MTA-treated teeth have a higher clinical success rate (including inflammatory response and dentin bridge formation) than those treated with Ca(OH)_2_ [10]. However, Ca(OH)_2_ treatment results in more dentin bridge formation and less inflammation compared with adhesive system treatment [10]. Interestingly, MTA- and calcium silicate-based materials have a comparable effect on dentin bridge formation, inflammatory response, and success rate [10]. Apart from these clinical observations, direct comparison of the effects of these materials on hDPs in vitro is limited. Thus, the aim of the present study was to compare the response of hDPs to four commercially available materials for vital pup therapy, DyCal^®^ (Ca(OH)_2_-based material), ProRoot^®^ MTA (MTA-based materials), Biodentine™ (calcium silicate-based material), and TheraCal™ LC (resin modified calcium silicate-based material). The effects of these materials on cytotoxicity, cell proliferation, cell migration, and odonto/osteogenic differentiation were examined.

## Methods

### Cell culture

The hDP isolation protocol was approved by the Human Research Ethics Committee, Faculty of Dentistry, Chulalongkorn University (No. 020/2018). Healthy permanent teeth extracted according to the dental treatment plan (impacted teeth) were collected from healthy donors [11–13]. Dental pulp tissues were separated from the teeth and cell isolation was performed by tissue explanation. The cells were maintained in Dulbecco’s Modified Eagle Medium (DMEM Gibco BRL, CA, USA) supplemented with 10% fetal bovine serum (FBS) (Gibco), 1% l-glutamine, 100 U/ml penicillin, and 100 μg/ml streptomycin (Gibco). The cells were incubated at 37 °C in a 5% CO_2_ humidified atmosphere. Cells obtained between passages 4‒6 were used in this study. To characterize the cells, the surface protein expression of hematopoietic and mesenchymal stem cell markers was examined using flow cytometry. In vitro mineralization by the cells was evaluated using alizarin red s and Von Kossa staining.

### Flow cytometry analysis

Cells were harvested using trypsin/EDTA solution to obtain a single cell suspension. The cells were immunostained in 1% horse serum (Gibco) in sterile phosphate buffered saline (PBS) with primary antibodies conjugated with fluorescent dye. The antibodies were PerCP-conjugated anti-CD45 (Immuno Tools, Friesoythe, Germany), FITC conjugated anti-human CD44 (BD Bioscience Pharmingen, NJ, USA), PE-conjugated anti-human CD105 (Immuno Tools), and APC-conjugated anti-human CD90 (Immuno Tools). Flow cytometry analysis was performed using a FACS^Calibur^ Flow cytometer (BD Bioscience, CA, USA).

### Materials and extraction medium preparation

DyCal^®^ and ProRoot^®^ MTA were purchased from Dentsply International Inc., DE, USA. Biodentine™ was purchased from Septodont, CO, USA. TheraCal™ LC was purchased from Bisco Inc., IL, USA. All materials were prepared following the manufacturers’ instructions into a cylindrical mold (5 mm high and 2.5 mm radius: 1.18 cm^2^ surface area). The mixtures were prepared in sterile condition and left for 24 h at room temperature before removing the mold. Culture medium (1 ml, following ISO 10993 part 12) was added to each material and incubated for 24 h at 37 °C in a 5% CO_2_ atmosphere. The extraction medium from each material was filtered (0.1 µm) and kept at − 20 °C until use. Each experiment employed the same batch of extraction medium.

In the odontogenic induction assay, the pulp capping materials were immersed in odontogenic medium. The odontogenic medium was prepared by adding 50 μg/ml ascorbic acid, 100 nM dexamethasone, and 5 mM β-glycerophosphate into the growth medium [11, 14]. The extraction medium in odontogenic medium was prepared the same as the growth medium. In the migration assay, serum-free culture medium was used to extract the materials.

### Cytotoxicity assay

An indirect cytotoxicity assay was performed using serially diluted extraction medium, according to the International Standard ISO (10993). Cells were plated into 96-well plate at 1 × 10^4^ cells per well and maintained in growth medium for 24 h. Serial dilutions of the extraction medium were prepared at 100, 50, 25, and 10% [15]. After 24 h exposure to the extraction medium, an MTT assay was performed and the percentage of cell number was calculated. Cells maintained in growth medium were used as control.

### Cell proliferation assay

Cells (1 × 10^4^ cells) were seeded in 24-well plates and maintained in growth medium for 24 h. Subsequently, the cells were exposed to each material’s extraction medium at 100, 50, 25, and 10% concentrations. At day 1, 4, and 7, cell viability was evaluated using an MTT assay. The percentage of cell number was calculated. The control condition was cells maintained in normal growth medium. The doubling time was calculated as previously described [16].

### Cell morphology evaluation

The effect of direct cell contact with the materials was evaluated. hDPs were directly seeded on the materials and maintained in normal growth medium. At 3, 6, 24, and 48 h, the cells were fixed with 3% glutaraldehyde (Sigma-Aldrich, MO, USA) in PBS for 30 min. The specimens were then dehydrated using a graded ethanol series, followed by hexamethyldisilazane (Sigma-Aldrich, MO, USA) treatment for 5 min. The samples were gold sputter-coated and observed using a Scanning Electron Microscope (SEM) (Quanta 250, FEI, Hillsboro, OR, USA).

### Migration assay

Cell migration was performed using an in vitro scratch assay. hDPs at a concentration of 2 × 10^5^ cells/well were seeded into 24-well plates and maintained in normal growth medium for 24 h. The culture medium was then replaced with serum-free culture medium and cultured for 24 h. A scratch was created using a sterilized-pipette tip and the cells were exposed to 25% of each material’s extracted medium. Images were captured using an inverted phase contrast microscope at the initial time and 24 h at the same location. Migrated cells were counted from at least 3 images from the same frame as the initial time image.

### ALP and in vitro mineralization assay

Cells were seeded into 24-well plates at a concentration of 5 × 10^4^ cells/well. After 24 h, the media were replaced with 25% of the different extracted osteogenic mediums and cultured for 14 days. Alkaline phosphatase staining (ALP) was performed at day 10, and Von Kossa and alizarin red s staining were performed at day 14 [11, 14]. For ALP staining, the cells were fixed and stained with TRACP & ALP double-stain kit (Takara Bio USA Inc., CA, USA). For alizarin red s staining, the cells were fixed with cold methanol and washed with deionized water. Alizarin red s solution (1% w/v) was incubated with the samples for 3 min, removed, and washed with DI water 3 times. The staining was solubilized with 10% cetylpyridinium chloride monohydrate (Sigma-Aldrich, MO, USA) solution and the absorbance was measured at 570 nm using a spectrophotometer. For Von Kossa staining, the cells were fixed with 4% paraformaldehyde (Sigma-Aldrich, MO, USA) in PBS for 10 min. After rinsing with deionized water, 5% silver nitrate (Sigma-Aldrich, MO, USA) was added and the samples were exposed to UV light for 60 min. The cells were washed and rinsed with 5% sodium thiosulfate (JT Baker) 3 times before counterstaining with methyl-green (Takara Bio USA Inc., CA, USA).

### Real-time polymerase chain reaction assay

Cells were seeded at concentration of 5 × 10^5^ cells per well in 24-well plated and maintained in growth medium for 24 h. The culture medium was then replaced with 25% of the different extracted medium and cultured for 4 and 10 days. For this experiment, the extraction medium from Biodentine™ and ProRoot^®^ MTA was prepared using growth medium (GM) or osteogenic induction medium (OM). Fresh growth medium was used as the control condition. To determine the mRNA expression of odonto/osteogenic differentiation markers, total RNA was extracted using Trizol reagent (RiboEx™, GeneAll^®^ Seoul, Korea) [17]. The RNA samples were converted to cDNA using a reverse transcriptase (Promega, WI, USA). FastStart^®^ Essential DNA Green Master was used to evaluate the expression level of osteogenic-related genes. The amplification profile was: 95 °C/20 s, 60 °C/20 s, and 72 °C/20 s for 45 cycles. The expression levels were normalized to *18S* expression levels and subsequently normalized to the control condition. Melting curve analysis was performed to determine product specificity. The oligonucleotide sequences used were *RUNX2* forward: 5′-ATGATGACACTGCCACCTCTG-3′, *RUNX2* Reverse: 5′-GGCTGGATAGTGCATTCGTG-3′, *DMP1* forward: 5′-CAGGAGCACAGGAAAAGGAG-3′, *DMP1* reverse: 5′-CTGGTGGTATCTTGGGCACT-3′, *DSPP* forward: 5′-ATATTGAGGGCTGGAATGGGGA-3′, *DSPP* reverse: 5′-TTTGTGGCTCCAGCATTGTCA-3′, *OCN* forward: 5′-CTTTGTGTCCAAGCAGGAGG-3′, *OCN* reverse: 5′-CTGAAAGCCGATGTGGTCAG-3′, and *18S* forward: 5′-GGCGTCCCCCAACTTCTTA-3′, reverse: 5′-GGGCATCACAGACCTGTTATT-3′.

### In vitro release of calcium ions

The in vitro release of calcium ions from the materials was investigated using a calcium detection kit (Calcium Colorimetric Assay, Sigma-Aldrich, MO, USA). The eluted medium samples were collected at 6 h, 1, 3, 5, and 7 days. The samples were placed in microtubes containing 100 µl of DMEM, and DMEM without materials was used as control [18]. The collected supernatants were stored at − 20 °C until analyzed.

### Statistical analysis

Each experiment performed at least three times. The results are presented as mean ± standard error of mean (SEM). Statistical analysis was performed using the Mann Whitney U test for two group comparison. Kruskal Wallis test followed by pairwise comparison was applied to those experiment comparing more than 3 groups. All statistical analyses and graphical illustration was performed using Prism 8 (GraphPad Software, CA, USA). Significance was considered at *p* < 0.05. All raw data was provided in the Additional file 1.

## Results

### Isolated cell characterization

**Fig. 1:**
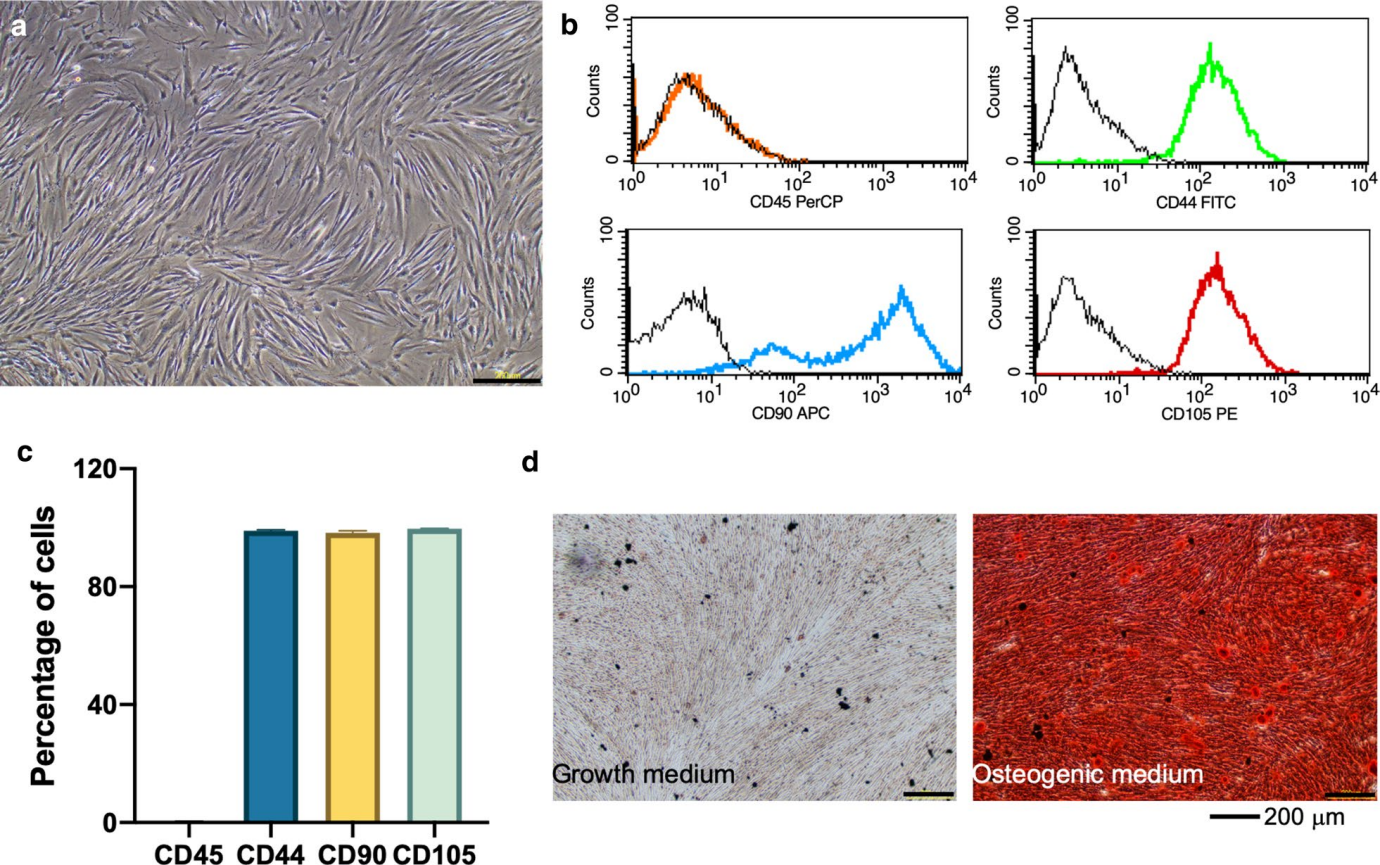
Characterization of the cells isolated from human dental pulp tissue. **a** Cell morphology was observed under an inverted phase contrast microscope. **b** Surface marker expression was investigated by flow cytometry analysis. **c** The percentage of cells expressing surface markers. **d** Alizarin red s staining was performed to identify mineral deposition after maintaining the cells in odontogenic medium for 14 days. Scale bars indicate 200 μm

Cells isolated from human dental pulp tissues (hDPs) exhibited a spindle shape and fibroblast-like morphology (Fig. 1a). These cells did not express CD45, a hematopoietic stem cell marker, but did express the mesenchymal stem cell markers CD44, CD90, and CD105 (Fig. 1b, c). To determine their differentiation ability, the cells were maintained in osteogenic medium for 14 days. Cells in normal growth medium were used as control. The results demonstrated that these cells deposited mineral crystals in vitro after induction, confirming their osteogenic differentiation potency (Fig. 1d).

### Cytotoxicity and proliferation assay

**Fig. 2:**
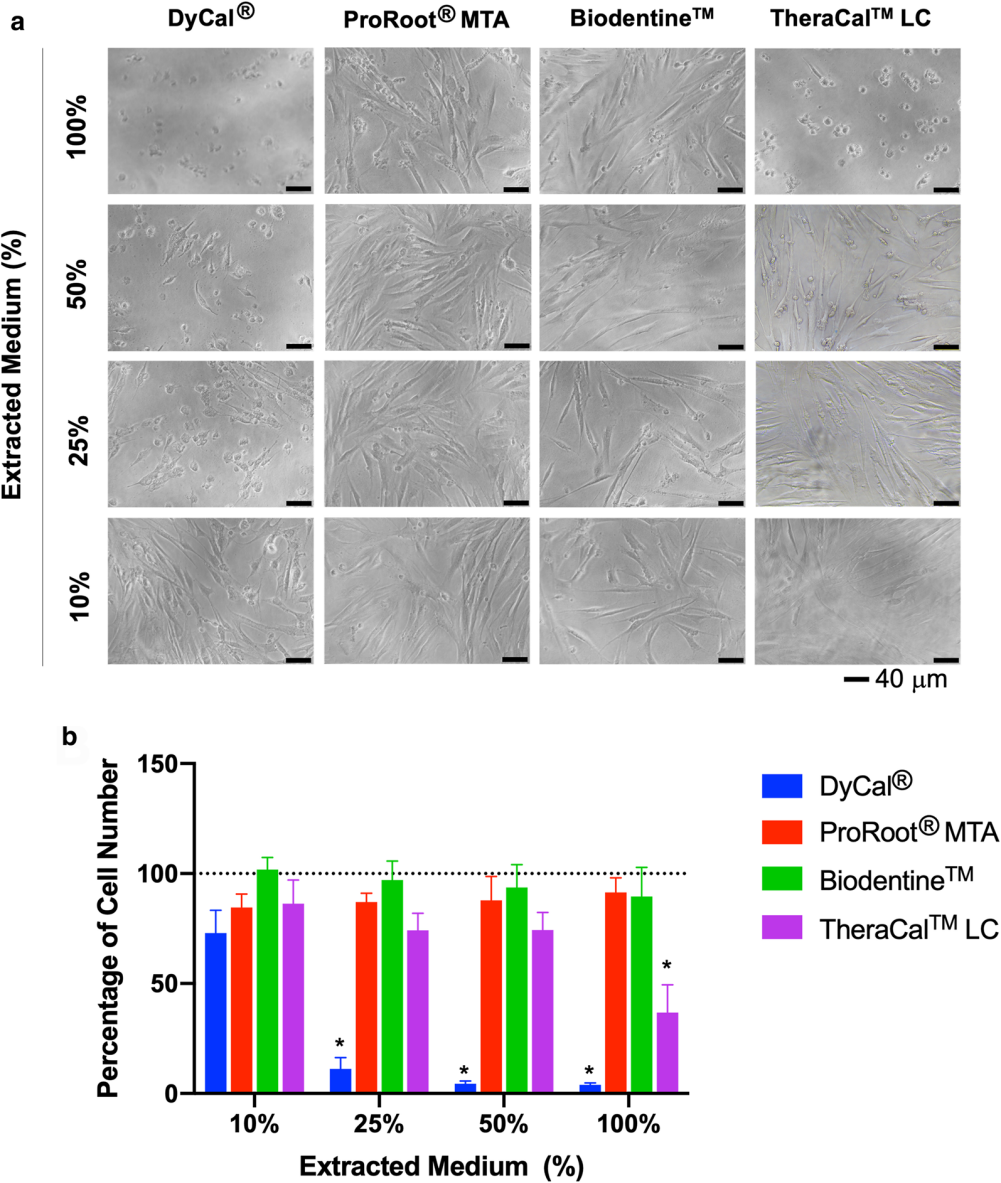
In vitro cytotoxicity of hDPs after exposure to extracted medium prepared from DyCal^®^, ProRoot^®^ MTA, Biodentine™ and TheraCal™ LC for 1 day. Cell cultured in normal growth medium was employed as the control. **a** Cell morphology was observed under an inverted phase contrast microscope 1 day after exposure. The hDPs were round and partial cell detachment was observed after exposure to the DyCal^®^ or TheraCal™ LC extraction medium. While fibroblast-like morphology shape was noted on those cells culture with ProRoot^®^ MTA or Biodentine™ extraction medium. **b** Cell viability was examined using MTT assay. The percentage of cell number. Dashed line indicates the reference value of the control condition. Asterisk designates significant differences compared with the control (Kruskal Wallis test followed by pairwise comparison, *p* < 0.05)

Cells were exposed to a range of concentrations of the extraction medium from DyCal^®^, ProRoot^®^ MTA, Biodentine™, and TheraCal™ LC for 24 h. Cell morphology was observed under an inverted phase contrast microscope (Fig. 2a) and cell viability was determined using an MTT assay (Fig. 2b). Cells maintained in normal growth medium were used as controls. The hDPs were round and partial cell detachment was observed after exposure to the DyCal^®^ extraction medium. A significant decrease in cell viability was observed in the cells treated with all DyCal^®^ extraction medium concentrations. Similarly, cells exposed to the TheraCal™ LC extraction medium were round in the 100% concentration and some detachment was noted at all concentrations. This observation corresponded with reduced cell viability. However, there was no significant difference in cell viability in the 10% TheraCal™ LC condition compared control. ProRoot^®^ MTA extraction medium at 10% and 25% caused the decrease of cell viability compared with the control. However, the cell viability was higher than 85% in those cells treated with 10% and 25% ProRoot^®^ MTA extraction medium. Biodentine™ extraction medium treatment resulted in a similar cytotoxicity to that of the controls.

**Fig. 3:**
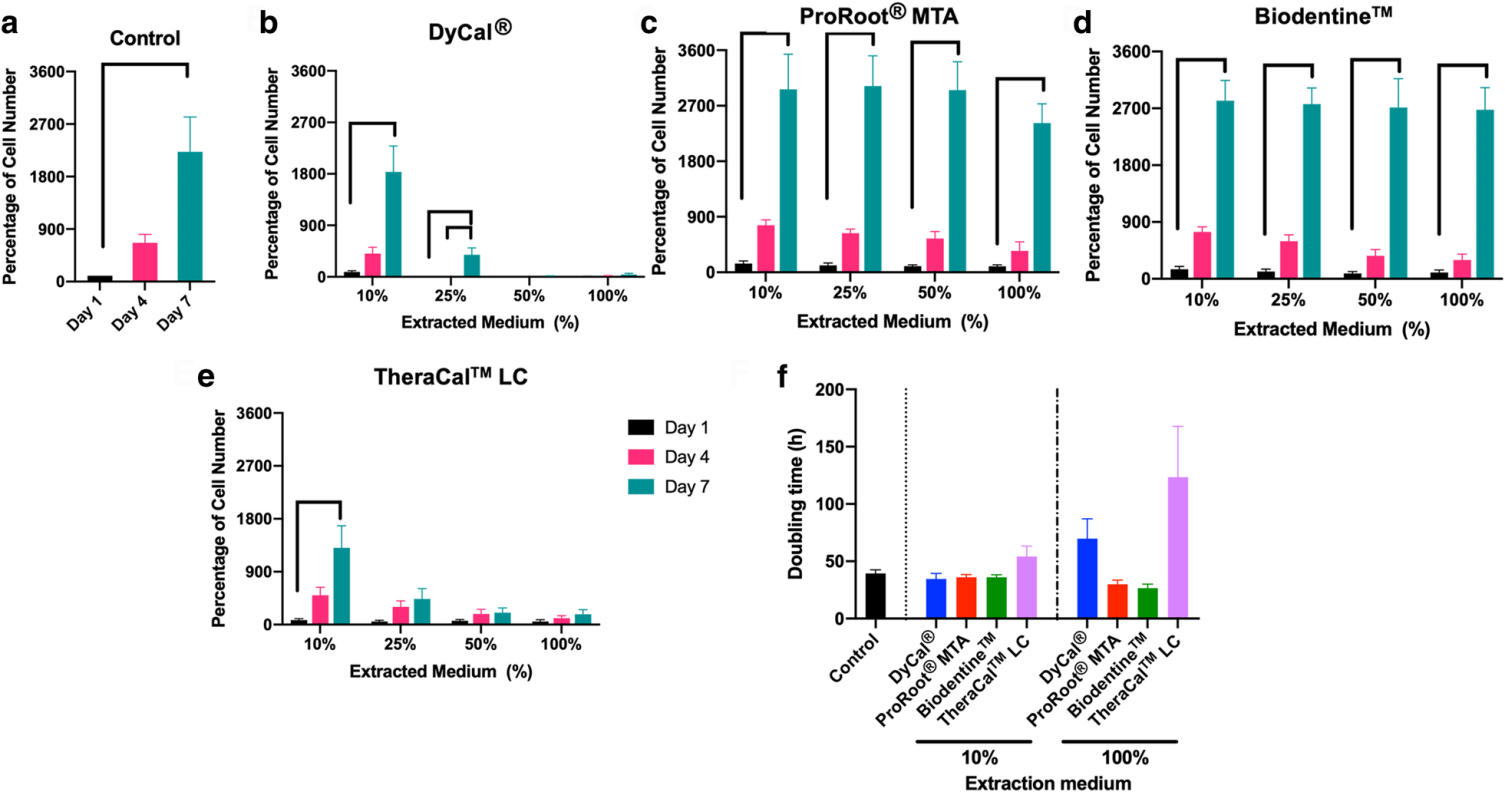
Cell proliferation after exposure to extracted medium. MTT assay was utilized to determine cell viability at day 1, 4 and 7. **a** The control condition was cells maintained in normal growth medium. The percentage of cell number at different time points after the hDPs were exposed to extraction medium from **b** DyCal^®^, **c** ProRoot^®^ MTA, **d** Biodentine™, and **e** TheraCal™ LC. **f** Doubling time was calculated using formula previously described. Bars indicate a significant difference (Kruskal Wallis test followed by pairwise comparison, *p* < 0.05)

In the proliferation assay, the hDPs were maintained in a range of concentration of extraction medium and cell viability was assessed at day 1, 4, and 7. Cells cultured in normal growth medium were employed as control (Fig. 3a). A significant increase in cell number percentage was observed at day 3 and 7 compared with day 1 in the control condition. At high percentages of DyCal^®^ and TheraCal™ LC extraction medium treatment, there was no marked increase in cell number percentage at later time points compared with day 1 (Fig. 3b, e). However, a significant increase in cell number percentage was observed at day 7 in the cells exposed to 10% DyCal^®^ and TheraCal™ LC extraction medium. When treated with ProRoot^®^ MTA or Biodentine™ extraction medium, the cells proliferated as determined by a significant increase in cell number percentage at later time points (Fig. 3c, d). These observations corresponded with the doubling time results (Fig. 3f). Treatment with 10% extraction medium from all four materials resulted in a comparable doubling time to that of the control. However, when treated with 100% extraction medium, the cells in the DyCal^®^ and TheraCal™ LC medium exhibited an increased doubling time compared with those in the ProRoot^®^ MTA or Biodentine™ medium and the control.

### Cell morphology observation

**Fig. 4:**
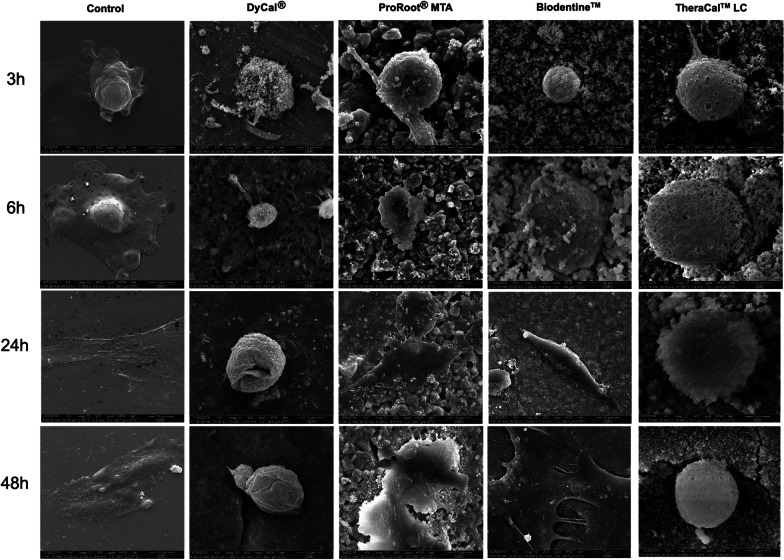
Representative SEM micrographs illustrate the morphology of hDPs directly seeded on DyCal^®^, ProRoot^®^ MTA, Biodentine™, and TheraCal™ LC at 3, 6, 24, and 48 h. Membrane blebbing and rupture were observed in DyCal^®^ group. Cells seeded on ProRoot^®^ MTA or Biodentine™ were able to attach and to spread on those materials. Cells on TheraCal™ LC exhibited membrane porosity

Cells were directly seeded on DyCal^®^, ProRoot^®^ MTA, Biodentine™, and TheraCal™ LC and maintained in normal culture medium. Glass cover slips were employed in the control condition. At 3, 6, 24, and 48 h, cell morphology was observed using SEM (Fig. 4). The cells attached and filopodia and lamellopodia were observed in the control condition at 3 h. Marked cell spreading was observed at 6 h and a completely flattened cell morphology was noted at 24 and 48 h. In the DyCal^®^ group, cell attachment was seen at 3 h. However, membrane blebbing and rupture were observed. The cells were not completely spread at 48 h. Cells seeded on ProRoot^®^ MTA or Biodentine™ demonstrated similar responses. The cells attached and spread on those materials. Complete cell spreading was noted at 24 and 48 h, comparable to the control condition. Lastly, the cells on TheraCal™ LC exhibited membrane porosity. However, some filopodia were observed. The cells were not completely spread on the material at 48 h.

### Cell migration assay

**Fig. 5:**
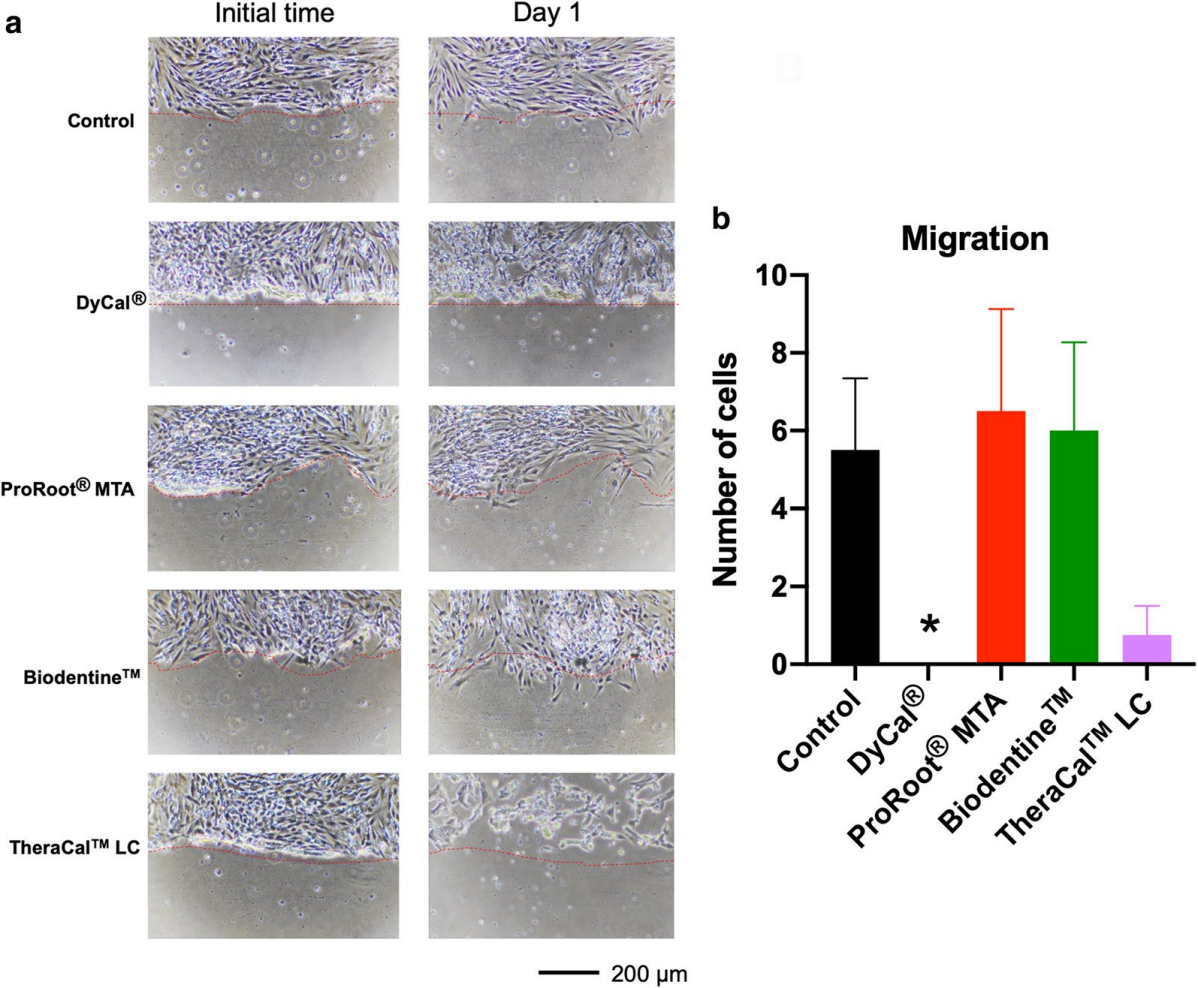
Cell migration was evaluated using a scratch assay. Cells were maintained in 25% extraction medium from the different materials. The control condition was cells maintained in normal growth medium. **a** Representative images of the scratch areas. The red dot line represented the initial border of the edge of scratched wound. At day 1, cell migration beyond the reference line was observed in the control, ProRoot^®^ MTA, and Biodentine™. **b** Graph illustrated the number of cells migrating from the edge at day 1. Asterisk designates significant differences compared with the control (Mann Whitney U test, *p* < 0.05)

An in vitro scratch assay was performed to evaluate cell migration. The hDPs were exposed to 25% serum-free extraction medium. Cells maintained in serum-free medium were employed as the control. At 24 h after creating the wound, cell migration was present in the control condition and in the ProRoot^®^ MTA or Biodentine™ extraction medium treated cells (Fig. 5a, b). However, cell migration was compromised in those cells exposed to DyCal^®^ or TheraCal™ LC medium.

### Odonto/osteogenic differentiation assay

**Fig. 6:**
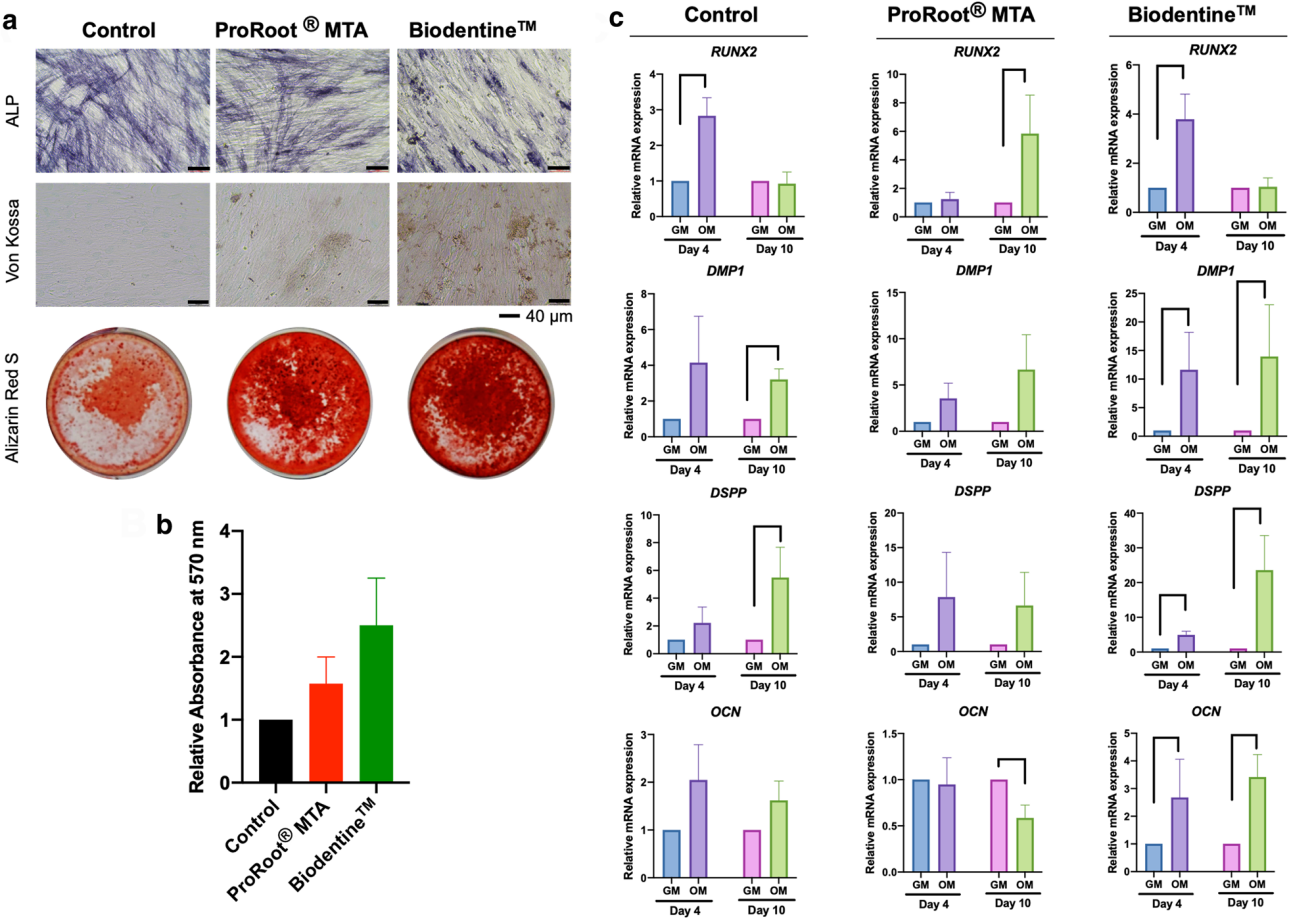
Effect of ProRoot^®^ MTA and Biodentine™ on odonto/osteogenic differentiation was evaluated. **a** ALP activity at day 10 after osteogenic induction and mineralization was examined using Von Kossa staining and alizarin red s staining at day 14. Scale bars indicate 40 μm. **b** The relative absorbance of the eluted alizarin red s dye. **c** The mRNA expression of odonto/osteogenic differentiation markers (*RUNX2*,* DMP1*,* DSPP*, and *OCN*) was examined using real-time polymerase chain reaction at day 4 and 10. Bars indicate a significant difference (Mann Whitney U test, *p* < 0.05)

Due to the in vitro toxicity of DyCal^®^ or TheraCal™, these materials were not employed in the differentiation study. Hence, only ProRoot^®^ MTA and Biodentine™ were investigated in this experiment. Extraction medium was prepared in both normal growth medium (GM) and osteogenic medium (OM). The cells were exposed to 25% extraction medium. ALP activity was determined at day 10 (Fig. 6a). There was no marked difference in ALP staining between these conditions. However, increased Von Kossa and alizarin red s staining at day 14 was observed in the cells cultured with ProRoot^®^ MTA and Biodentine™ extraction medium (Fig. 6a). The alizarin red s staining was solubilized and the quantitative analysis was performed by measuring the optical density of dissoluted solution. The quantitative values were plotted in Fig. 6b.

The mRNA expression of odonto/osteogenic markers was evaluated at day 4 and 10 after induction. The expression pattern of the cells treated with Biodentine™ extraction medium was similar to the control. *RUNX2* expression levels were increased at day 4 in osteogenic medium compared with the control and subsequently decreased at day 10 (Fig. 6c). The upregulation of *DMP1, DSPP,* and *OCN* expression was observed at day 4 and day 10 in the control and Biodentine™ condition. However, the cells in the control condition exhibited a significant increase in *DMP1* and *DSPP* expression at day 10, while those in Biodentine™ extraction medium demonstrated a significant increase in *DMP1, DSPP,* and *OCN* mRNA expression at all time points. The ProRoot^®^ MTA treatment resulted in a significant upregulation in *RUNX2* expression at day 10. Interestingly, the *OCN* mRNA expression was not significantly different in osteogenic medium compared with the growth medium control at day 4, however, a marked decrease was noted at day 10.

### Calcium release assay

**Fig. 7:**
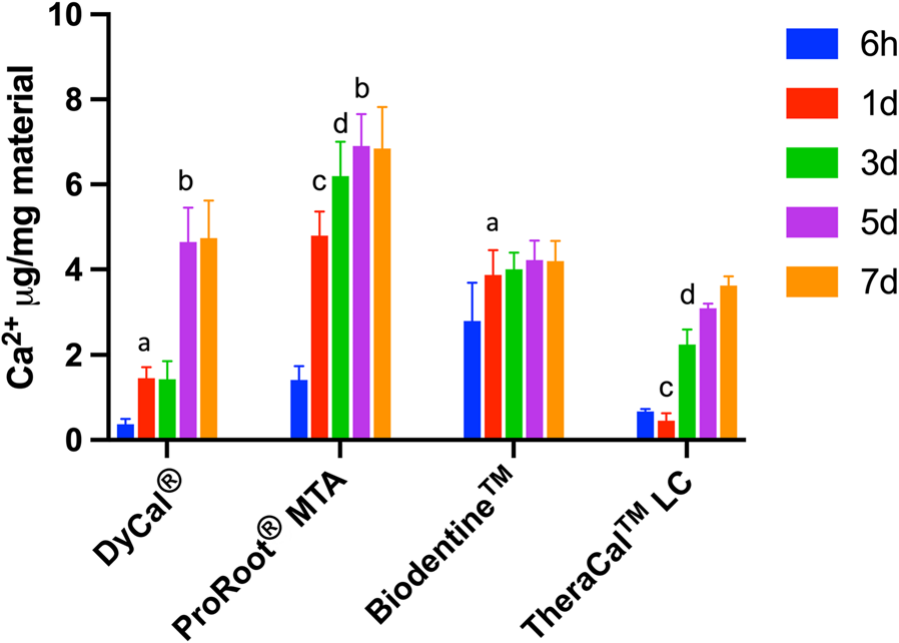
The release of calcium ions from DyCal^®^, ProRoot^®^ MTA, Biodentine™, and TheraCal™ LC was quantified at 6 h, 1, 3, 5, and 7 days. Letters indicated the statistically significant difference (Kruskal Wallis test followed by pairwise comparison, *p* < 0.05)

All materials contained calcium as a component (data not shown) and released calcium ions (Fig. 7). At 6 h, the calcium ions released from Biodentine™ was the highest. In all materials, the amount of released calcium at 5 and 7 days was similar for each material when distinctly evaluated. Moreover, the released calcium ions increased in a time-dependent manner in the DyCal^®^, ProRoot^®^ MTA, and TheraCal™ LC groups. However, the level of calcium ions released from Biodentine™ was comparable between all time-points. At day 7, ProRoot^®^ MTA released the highest amount of calcium ions compared with other materials.

## Discussion

In the past decade, many commercial products have become available as direct pulp capping materials. Apart from clinical success reports, the biological mechanism by which these materials stimulate tertiary dentin formation remains unclear. Many quantitative and qualitative assessments of in vitro and in vivo toxicity show the effectiveness of pulp capping materials. The investigations were usually carried out to gain increased understanding of the biological mechanisms of tertiary dentin formation. However, the limitations of those reports were the types of tested cells, exposure duration and formulation of the materials. Studies directly comparing the different materials are limited. The present study employed DyCal^®^, ProRoot^®^ MTA, Biodentine™, and TheraCal™ LC as representatives of the current clinically used materials.

The present study demonstrated that DyCal^®^ and TheraCal™ LC were cytotoxic in vitro while ProRoot^®^ MTA and Biodentine™ demonstrated the better biocompatibility to hDPs. These findings are comparable to previous studies in stem cells isolated from human exfoliated deciduous teeth (SHEDs), periodontal ligament cells (PDLs), and a mouse dental pulp cell line (MDPC-23) [14, 19–25]. However, the present study illustrated that treatment with 10% extracted medium from each material resulted in the 70% or above of cytotoxicity levels compared with the control. In addition, the cells proliferated in the 10% concentrations. These outcomes were comparable to previous reports demonstrating that ProRoot^®^ MTA, Biodentine™, and TheraCal™ LC were not cytotoxic or genotoxic to human dental pulp cells at concentrations of 0‒1000 μg/ml [22]. Recently, report demonstrated that nonresident bone marrow derived cells participate in reparative dentin formation after treating with ProRoot^® ^MTA and Biodentine™ [26]. In this regard, the nonresident bone marrow derived cells were found in associate with reparative dentin and these cells expressed dentin sialoprotein, implicating the involvement of nonresident bone marrow derived cells in tertiary dentin formation [26].

The cell death that occurred from the DyCal^®^ treatment may be due to increased pH. This increase was observed from the color change of the pH indicator in the culture medium. Correspondingly, DyCal^®^ produced pH 10.15 at 3 h and pH increased to 10.88 at 24 h after setting [27]. The high release of hydroxyl ions leads to cell death because disrupted membrane morphology was observed on the SEM micrographs [23, 25]. The cytotoxicity of TheraCal™ LC may be due to remaining unpolymerized resin monomers. However, when using the appropriate curing technique, there should be no unpolymerized monomers. Further, the cured TheraCal™ LC exhibited very low pore volume percentage per total volume (0.19 ± 0.09%) [28]. Hence, the effect of unpolymerized resin monomers on cell death activation is unlikely. However, previous studies demonstrated that cured TheraCal™ LC released specific additives, camphoroquinone and ethyl-4-(dimethylamino)benzoate [28]. Human dental pulp fibroblasts treated with camphoroquinone demonstrated increased reactive oxygen species production [29, 30]. Taken together, the light curing additives released from TheraCal™ LC might induce cell death by increasing the production of reactive oxygen species; while the high pH produced by DyCal^®^ provides a high concentration of hydroxyl ions and subsequently causes cell death.

The present study demonstrated that ProRoot^®^ MTA and Biodentine™ at 100% extraction medium was not toxic to hDPs. Sequeira et al. reported that ProRoot^®^ MTA did not affect apical papilla cell viability at all extraction medium concentrations, while 100% Biodentine™ extraction medium induced cell death [31]. Unlike DyCal^®^, ProRoot^®^ MTA does not increase the production of reactive oxygen species, nitric oxide, or prostaglandin E2 in immortalized human dental pulp cells [32]. TheraCal™ LC but not Biodentine™ induced IL-8 release from dental pulp cells [33]. TheraCal™ LC also induced cell apoptosis higher than MTA and Biodentine™ [34]. Another hypothesis is that TheraCal™ LC decreased cellular metabolic activity [35]. These mechanisms could partly explain the different cytotoxic effect among the tested materials.

The present study demonstrated that ProRoot^®^ MTA slightly reduced cell viability at 10% and 25% extraction medium while hDPs exposed to higher concentration of ProRoot^®^ MTA extraction medium did not exhibit the marked difference of cell viability compared with the control. Another study demonstrated that ProRoot^®^ MTA was cytotoxic to human dental pulp fibroblasts at all extraction medium concentration at 24, 48, and 72 h [36]. These disparate results might be due to different cell types and extraction methods. The present study used the ISO 10993 protocol. Thus, standardized methods for extraction medium preparation were employed. Therefore, the in vitro evaluation of the cell responses to these materials could be compared between studies.

It has been reported that pulp capping materials solubilize dentin, leading to the release of various growth factors that promote several biological events [37], particularly cell migration, that enhance pulp healing. In the present study, serum-free culture medium was used for extracting the materials in the in vitro scratch wound assay. Thus, the influence of serum proteins and released factors from dentin were excluded. We found that ProRoot^®^ MTA and Biodentine™ extraction medium promoted cell migration at 24 h, similar to the controls. Similarly, a study evaluating cell migration in serum-free medium using a transwell migration assay demonstrated that ProRoot^®^ MTA and Biodentine™ promoted the migration of human bone marrow-derived mesenchymal stem cells and hDPs, respectively [38, 39]. The ProRoot^®^ MTA and Biodentine™ extraction medium in DMEM supplemented with 20% fetal bovine serum promoted a similar rate of cell migration at 24 h compared with the control [31]. However, at 48 h, complete scratch wound healing in vitro was observed in the control, but not in the ProRoot^®^ MTA and Biodentine™ treated groups [31]. The marked decrease in cell migration in the DyCal^®^ or TheraCal™ LC treated groups could be due to the effect of these materials on cell viability. Our cell migration results correspond with our cell attachment and spreading results. In general, cells have to attach and spread on a surface and subsequently migrate. Cell attachment and spreading were compromised in the DyCal^®^ or TheraCal™ LC treated groups as observed by SEM. Hence, their migration would also be affected.

Despite these negative in vitro effects, the successful application of DyCal^®^ and TheraCal™ LC in direct pulp capping treatment has been reported. The clinical success rate at a 6 month follow-up of DyCal^®^ and TheraCal™ LC was approximately 73% and 66%, respectively, which were not significantly different [40]. Increased dentin bridge formation was observed in direct pulp capping with TheraCal™ LC compared with DyCal^®^, however, the difference was not significant [41, 42]. Due to the complex cell responses, an in vivo study is required to confirm the effect of these pulp capping materials on tertiary dentin formation. However, an in vitro investigation could allow for in-depth evaluation of the mechanism(s) of the cell responses to these materials.

A systematic review and meta-analysis indicated that MTA and calcium silicate materials exhibited a similar clinical success rate, dentin bridge formation, and inflammatory response [10]. The present study demonstrated that ProRoot^®^ MTA and Biodentine™ treatment slightly enhanced in vitro mineralization and osteogenic marker gene expression compared with normal osteogenic medium. In parallel with previous reports, ProRoot^®^ MTA enhanced mineral deposition compared with control in immortalized human dental pulp cells, and human mandibular derived MSCs and stem cells isolated from apical papilla (SCAPs) [32, 43, 44]. Biodentine™ treatment also resulted in increased mineralization by SCAPs and human mandibular derived MSCs [43, 44]. The different cell types and more importantly, extraction medium preparation methods could influence different cell responses. Hence, standardized methods should be used for extraction medium preparation to allow for direct comparison between studies.

It is hypothesized that the tricalcium and dicalcium silicate in ProRoot^®^ MTA and Biodentine™ are key factors in generating cell responses [45]. Calcium ions are considered as a bioactive ingredient in pulp capping materials. In mouse bone marrow mesenchymal stem cells, elevated extracellular calcium ions promoted cell proliferation and *Fgf2*,* Tgfb1*, and *Opn* mRNA expression [46]. The elevated Opn level influenced cell migration, but not cell proliferation or mineralization [46]. In hDPs, increased extracellular calcium ions resulted in cell apoptosis and increased mineralization [47, 48]. It was hypothesized that the increase in cell apoptosis at early calcium ion treatment time points in hDPs may be associated with the early onset of mineral deposition by these cells [47]. It has been reported in vascular smooth muscle cells that apoptotic bodies may involve in calcium ions concentration and subsequently promoted the onset of calcification [49]. In other words, apoptotic bodies function as nucleator for calcium and phosphate precipitation [50]. However, calcium ions did not markedly influence hDP cell proliferation in vitro [47, 48]. Similar to mesenchymal stem cells, calcium ions promoted *OPN* mRNA expression in hDPs at early time points [47]. However, the long-term supplementation of calcium ions in osteogenic medium (14–21 days) resulted in significantly decreased *OPN* mRNA levels, despite increased mineral deposition [48]. The link between *OPN* expression in calcium ions-treated hDPs and other biological functions, such as those in mesenchymal stem cells, has not yet been established. Further, reduced *RUNX2* and *COL1A2* mRNA levels, but increased *OCN* expression, was observed in the elevated extracellular calcium ion condition compared with the control [48]. The present study illustrated that *RUNX2* and *OCN* mRNA levels were increased in the Biodentine™ treated groups, while only *RUNX2* expression was increased in the ProRoot^®^ MTA treated group. These observations suggest that calcium ions are not solely responsible in the biological effects of these materials. Thus, the bioactive components of these pulp capping materials should be further evaluated to better understand the basic mechanism(s) of dental pulp responses.

## Conclusions

The present study described the biological responses of hDPs to various direct pulp capping materials in vitro. DyCal^®^ and TheraCal™ LC were found to be toxic to the cells. Cell attachment, spreading, proliferation, and migration were compromised when the cells were exposed to DyCal^®^ or TheraCal™ LC. In contrast, ProRoot^®^ MTA and Biodentine™ exhibited biocompatibility and supported cell activities toward regeneration potency. We show here that, despite their clinical success, the in vitro biological effects and molecular mechanisms should be further investigated to clarify the contribution of these bioactive materials to reparative dentin formation and dental pulp vitality protection. Further study is needed.

## Supplementary information

**Additional file 1:** Raw data used in the article.

## Data Availability

The datasets generated during and analyzed during the current study are available in supplementary file. Other images are available from the corresponding author on reasonable request.

## Abbreviations

ALP: Alkaline phosphatase staining
hDPs: Human dental pulp stem cells
MTA: Mineral trioxide aggregate
SEM: Scanning electron microscope
SHEDs: Human exfoliated deciduous teeth
PDLs: Periodontal ligament cells

## References

[1] Wells C, Dulong C, McCormack S. Vital pulp therapy for endodontic treatment of mature teeth: a review of clinical effectiveness, cost-effectiveness, and guidelines. Ottawa (ON): Canadian Agency for Drugs and Technologies in Health 2019 Jul. https://www.ncbi.nlm.nih.gov/books/NBK546327/. 31525010

[2] Goldberg M (2011). Pulp healing and regeneration: more questions than answers. Adv Dent Res.

[3] Téclès O, Laurent P, Zygouritsas S, Burger A-S, Camps J, Dejou J (2005). About I: activation of human dental pulp progenitor/stem cells in response to odontoblast injury. Arch Oral Biol.

[4] Shi S, Gronthos S (2003). Perivascular niche of postnatal mesenchymal stem cells in human bone marrow and dental pulp. J Bone Miner Res.

[5] Gronthos S, Mankani M, Brahim J, Robey PG, Shi S (2000). Postnatal human dental pulp stem cells (DPSCs) in vitro and in vivo. Proc Natl Acad Sci USA.

[6] Osathanon T, Nowwarote N, Pavasant P (2011). Basic fibroblast growth factor inhibits mineralization but induces neuronal differentiation by human dental pulp stem cells through a FGFR and PLCγ signaling pathway. J Cell Biochem.

[7] Osathanon T, Sawangmake C, Nowwarote N, Pavasant P (2014). Neurogenic differentiation of human dental pulp stem cells using different induction protocols. Oral Dis.

[8] Sawangmake C, Nowwarote N, Pavasant P, Chansiripornchai P, Osathanon T (2014). A feasibility study of an in vitro differentiation potential toward insulin-producing cells by dental tissue-derived mesenchymal stem cells. Biochem Biophys Res Commun.

[9] Yen AH-H, Sharpe PT (2008). Stem cells and tooth tissue engineering. Cell Tissue Res.

[10] Paula AB, Laranjo M, Marto C-M, Paulo S, Abrantes AM, Casalta-Lopes J, Marques-Ferreira M, Botelho MF, Carrilho E (2018). Direct pulp capping: What is the most effective therapy?—Systematic review and meta-analysis. J Evid Based Dent Pract.

[11] Manokawinchoke J, Nattasit P, Thongngam T, Pavasant P, Tompkins KA, Egusa H, Osathanon T (2017). Indirect immobilized Jagged1 suppresses cell cycle progression and induces odonto/osteogenic differentiation in human dental pulp cells. Sci Rep.

[12] Manokawinchoke J, Ritprajak P, Osathanon T, Pavasant P (2016). Estradiol induces osteoprotegerin expression by human dental pulp cells. Odontology.

[13] Manaspon C, Boonprakong L, Porntaveetus T, Osathanon T (2020). Preparation and characterization of Jagged1-bound fibrinogen-based microspheres and their cytotoxicity against human dental pulp cells. J Biomater Appl.

[14] Wang F, Okawa H, Kamano Y, Niibe K, Kayashima H, Osathanon T, Pavasant P, Saeki M, Yatani H, Egusa H (2015). Controlled osteogenic differentiation of mouse mesenchymal stem cells by tetracycline-controlled transcriptional activation of amelogenin. PLoS ONE.

[15] Pedano MS, Li X, Li S, Sun Z, Cokic SM, Putzeys E, Yoshihara K, Yoshida Y, Chen Z, Van Landuyt K (2018). Freshly-mixed and setting calcium-silicate cements stimulate human dental pulp cells. Dent Mater.

[16] Osathanon T, Subbalekha K, Sastravaha P, Pavasant P (2012). Notch signalling inhibits the adipogenic differentiation of single-cell-derived mesenchymal stem cell clones isolated from human adipose tissue. Cell Biol Int.

[17] Charoenpong H, Osathanon T, Pavasant P, Limjeerajarus N, Keawprachum B, Limjeerajarus CN, Cheewinthamrongrod V, Palaga T, Lertchirakarn V, Ritprajak P (2019). Mechanical stress induced S100A7 expression in human dental pulp cells to augment osteoclast differentiation. Oral Dis.

[18] Chung CJ, Kim E, Song M, Park J-W, Shin S-J (2016). Effects of two fast-setting calcium-silicate cements on cell viability and angiogenic factor release in human pulp-derived cells. Odontology.

[19] Farhadmollashahi N, Ghotbi F, Karkeabadi H, Havaei R (2016). Cytotoxic effects of mineral trioxide aggregate, calcium enrichedmixture cement, Biodentine and octacalcium pohosphate onhuman gingival fibroblasts. J Dent Res Dent Clin Dent Prospects.

[20] Collado-González M, García-Bernal D, Oñate-Sánchez R, Ortolani-Seltenerich P, Álvarez-Muro T, Lozano A, Forner L, Llena C, Moraleda J, Rodríguez-Lozano F (2017). Cytotoxicity and bioactivity of various pulpotomy materials on stem cells from human exfoliated primary teeth. Int Endod J.

[21] Escobar-García DM, Aguirre-López E, Méndez-González V, Pozos-Guillén A (2016). Cytotoxicity and initial biocompatibility of endodontic biomaterials (MTA and Biodentine^TM^) used as root-end filling materials. Biomed Res Int.

[22] Zakerzadeh A, Esnaashari E, Dadfar S (2017). In vitro comparison of cytotoxicity and genotoxicity of three vital pulp capping materials. Iran Endod J.

[23] Poggio C, Ceci M, Dagna A, Beltrami R, Colombo M, Chiesa M (2015). In vitro cytotoxicity evaluation of different pulp capping materials: a comparative study. Arh Hig Rada Toksikol.

[24] Loison-Robert LS, Tassin M, Bonte E, Berbar T, Isaac J, Berdal A, Simon S, Fournier BP (2018). In vitro effects of two silicate-based materials, Biodentine and BioRoot RCS, on dental pulp stem cells in models of reactionary and reparative dentinogenesis. PLoS ONE.

[25] Arandi NZ, Rabi T (2018). TheraCal LC: from biochemical and bioactive properties to clinical applications. Int J Dent.

[26] Frozoni M, Marques MR, Hamasaki SK, Mohara NT, de Jesus SA, Zaia AA (2020). Contribution of bone marrow-derived cells to reparative dentinogenesis using bone marrow transplantation model. J Endod.

[27] Luczaj-Cepowicz E, Marczuk-Kolada G, Pawinska M, Obidzinska M, Holownia A (2017). Evaluation of cytotoxicity and pH changes generated by various dental pulp capping materials—an in vitro study. Folia Histochem Cytobiol.

[28] Nilsen BW, Jensen E, Örtengren U, Michelsen VB (2017). Analysis of organic components in resin-modified pulp capping materials: critical considerations. Eur J Oral Sci.

[29] Engelmann J, Volk J, Leyhausen G, Geurtsen W (2005). ROS formation and glutathione levels in human oral fibroblasts exposed to TEGDMA and camphorquinone. J Biomed Mater Res B Appl Biomater.

[30] Atsumi T, Ishihara M, Kadoma Y, Tonosaki K, Fujisawa S (2004). Comparative radical production and cytotoxicity induced by camphorquinone and 9-fluorenone against human pulp fibroblasts. J Oral Rehabil.

[31] Sequeira DB, Seabra CM, Palma PJ, Cardoso AL, Peça J, Santos JM (2018). Effects of a new bioceramic material on human apical papilla cells. J Funct Biomater.

[32] Chang S-W, Lee S-Y, Kum K-Y, Kim E-C (2014). Effects of ProRoot MTA, bioaggregate, and micromega MTA on odontoblastic differentiation in human dental pulp cells. J Endod.

[33] Jeanneau C, Laurent P, Rombouts C, Giraud T, About I (2017). Light-cured tricalcium silicate toxicity to the dental pulp. J Endod.

[34] Collado-Gonzalez M, Garcia-Bernal D, Onate-Sanchez RE, Ortolani-Seltenerich PS, Alvarez-Muro T, Lozano A, Forner L, Llena C, Moraleda JM, Rodriguez-Lozano FJ (2017). Cytotoxicity and bioactivity of various pulpotomy materials on stem cells from human exfoliated primary teeth. Int Endod J.

[35] Hebling J, Lessa FC, Nogueira I, Carvalho RM, Costa CA (2009). Cytotoxicity of resin-based light-cured liners. Am J Dent.

[36] Zhu L, Yang J, Zhang J, Peng B (2014). A comparative study of BioAggregate and ProRoot MTA on adhesion, migration, and attachment of human dental pulp cells. J Endod.

[37] Tomson P, Lumley P, Smith A, Cooper P (2017). Growth factor release from dentine matrix by pulp-capping agents promotes pulp tissue repair-associated events. Int Endod J.

[38] Sun Y, Liu J, Luo T, Shen Y, Zou L (2019). Effects of two fast-setting pulp-capping materials on cell viability and osteogenic differentiation in human dental pulp stem cells: an in vitro study. Arch Oral Biol.

[39] D'Antò V, Di Caprio MP, Ametrano G, Simeone M, Rengo S, Spagnuolo G (2010). Effect of mineral trioxide aggregate on mesenchymal stem cells. J Endod.

[40] Cengiz E, Yilmaz HG (2016). Efficacy of erbium, chromium-doped: yttrium, scandium, gallium, and garnet laser irradiation combined with resin-based tricalcium silicate and calcium hydroxide on direct pulp capping: a randomized clinical trial. J Endod.

[41] Parolia A, Kundabala M, Rao N, Acharya S, Agrawal P, Mohan M, Thomas M (2010). A comparative histological analysis of human pulp following direct pulp capping with Propolis, mineral trioxide aggregate and Dycal. Aust Dent J.

[42] Cannon M, Gerodias N, Vieira A, Percinoto C, Jurado R (2014). Primate pulpal healing after exposure and TheraCal application. Int J Clin Pediatr Dent.

[43] Margunato S, Taşlı PN, Aydın S, Kazandağ MK, Şahin F (2015). In vitro evaluation of ProRoot MTA, Biodentine, and MM-MTA on human alveolar bone marrow stem cells in terms of biocompatibility and mineralization. J Endod.

[44] Saberi E, Farhad-Mollashahi N, Aval FS, Saberi M (2019). Proliferation, odontogenic/osteogenic differentiation, and cytokine production by human stem cells of the apical papilla induced by biomaterials: a comparative study. Clin Cosmet Investig Dent.

[45] Camilleri J (2008). The chemical composition of mineral trioxide aggregate. J Conserv Dent.

[46] Lee MN, Hwang H-S, Oh S-H, Roshanzadeh A, Kim J-W, Song JH, Kim E-S, Koh J-T (2018). Elevated extracellular calcium ions promote proliferation and migration of mesenchymal stem cells via increasing osteopontin expression. Exp Mol Med.

[47] An S, Gao Y, Huang Y, Jiang X, Ma K, Ling J (2015). Short-term effects of calcium ions on the apoptosis and onset of mineralization of human dental pulp cells in vitro and in vivo. Int J Mol Med.

[48] An S, Gao Y, Ling J, Wei X, Xiao Y (2012). Calcium ions promote osteogenic differentiation and mineralization of human dental pulp cells: implications for pulp capping materials. J Mater Sci Mater Med.

[49] Proudfoot D, Skepper JN, Hegyi L, Farzaneh-Far A, Shanahan CM, Weissberg PL (2001). The role of apoptosis in the initiation of vascular calcification. Z Kardiol.

[50] Proudfoot D, Skepper JN, Hegyi L, Bennett MR, Shanahan CM, Weissberg PL (2000). Apoptosis regulates human vascular calcification in vitro: evidence for initiation of vascular calcification by apoptotic bodies. Circ Res.

